# Demographic risk factors for extra-pulmonary tuberculosis among adolescents and adults in Saudi Arabia

**DOI:** 10.1371/journal.pone.0213846

**Published:** 2019-03-27

**Authors:** Hawra Al-Ghafli, Bright Varghese, Mushira Enani, Abdulrahman Alrajhi, Sameera Al Johani, Ali Albarrak, Sahar Althawadi, Noura Elkizzi, Sahal Al Hajoj

**Affiliations:** 1 Department of Infection and Immunity, King Faisal Specialist Hospital and Research Centre, Riyadh, Saudi Arabia; 2 Medical Specialties Department, King Fahad Medical City, Riyadh, Saudi Arabia; 3 Department of Medicine, King Faisal Specialist Hospital and Research Centre, Riyadh, Saudi Arabia; 4 Department of Microbiology, King Abdul Aziz Medical City, Riyadh, Saudi Arabia; 5 Department of Medicine, Prince Sultan Military Medical City, Riyadh, Saudi Arabia; 6 Department of Pathology and Laboratory Medicine, King Faisal Specialist Hospital and Research Centre, Riyadh, Saudi Arabia; 7 Department of Microbiology, Prince Sultan Military Medical City, Riyadh, Saudi Arabia; 8 College of Medicine, Alfaisal University, Riyadh, Saudi Arabia; Institute of Medical Sciences, Banaras Hindu University, INDIA

## Abstract

Despite low infectious potential of extrapulmonary tuberculosis (EPTB), it poses significant clinical challenges in terms of diagnosis and treatment monitoring. Understanding the main demographical risk factors for disease characteristics of EPTB plays a crucial role in speeding up diagnosis process and improving overall clinical experience. The aim of this study was to investigate the main demographical and clinical risk factors for EPTB among adults and adolescents for the first time in Saudi Arabia. A cross-sectional multicenter study was carried out on a collection of 902 extrapulmonary *Mycobacterium tuberculosis* complex (MTBC) isolates with demographical and clinical data. All isolates were subjected to spoligotyping and 24-loci based MIRU-VNTR typing. The association between two potential variables was assessed using odd ratios (OR) calculations. Independent risk factors for EPTB and diseases characteristics of EPTB were identified using multivariate regression model analyses. Gender was found to be significantly associated with lymph node, gastrointestinal, central nervous system and urogenital TB. Lymph node TB showed statistical association to age group below 25 years, non-Saudis and South East Asian ethnicity. While gastrointestinal TB demonstrated an association with patients above 60 years old, and Saudis. Multivariate analysis showed that gender is an independent risk factor to urogenital TB (p 0.03) and lymph node TB (p 0.005). On the other hands, South Asian (p 0.01) and South East Asian (p 0.03) ethnicities were both identified as independent risk factors significantly associated with EPTB. MTBC lineages, site of infections, gender, HIV and smear positivity showed no significant association. Nationwide qualitative-studies are highly warranted in the future to further understand the main demographic risk factors for disease characteristics of EPTB.

## Introduction

Tuberculosis (TB) remains an enormous public health threat [1]. Etiological agents of TB, members of *Mycobacterium tuberculosis* complex (MTBC), primarily invade the host respiratory tract with majority of TB patients being diagnosed with active pulmonary TB (PTB) infections. However, compromised host-immune system (e.g. due to synergism of HIV and MTB infections) increases the risk of more serious TB manifestations such as concurrent extra-pulmonary TB (EPTB) (a clinical presentation of TB that entitles the dissemination of mycobacterial bacilli in both pulmonary and extra-pulmonary sites). This is largely due to failure of host-immune system to enclose *Mycobacterium tuberculosis* (MTB) bacilli within lung parenchyma. EPTB diseases may emerge at one particular body site or at multiple body sites even with no sign of PTB-concurrency (e.g due to re-activation of previous TB infection). Non-concurrent EPTB is very challenging at diagnosis and treatment, albeit of its lower infectious potential. Hence, investigating the main risk factors associated with EPTB clinical phenotype is a crucial step towards speeding up diagnosis process and improving overall clinical experience for patients.

Several studies have found a significant impact of gender and age on the development of EPTB among active TB patients. For instance, a good number of reports have shown that women are more likely to present their MTB infections as EPTB compared to men [2–4]. Similarly, a recent population-based study from Taiwan evaluated the effect of age and gender on susceptibility to TB disease and stated that older women (aged 45 years and above) have higher risk of developing concurrent EPTB with PTB compared to men [5]. This was suspected to be due to weaker immune status, hormonal changes, nutritional status, socio-economic and cultural factors experienced by older women [4, 6, 7]. In addition, an increasing number of studies have also reported on host-pathogen interaction and clinical presentation of TB. Whilst a segment of these publications concluded that host-ethnicity related factors play a critical role in determining TB clinical phenotype [5, 8, 9], others suggested that MTBC lineages could be the main detriments of TB clinical presentation [10–12]. It is worth mentioning that these findings remain to a large extent inconsistent and variable from one population to another, urging for similar representative studies from each setting.

In Saudi Arabia, classified as a moderate TB-burden country with an incidence rate of 25 per 100,000 populations per year, bacterial and demographical determinants of EPTB have not been fully investigated. To start with, a previous national report showed that the rate of recorded EPTB incidence ranged from 25% to 31%; the reported rate is certainly higher than that in developed countries [13–16]. High migration dynamics due to workforce infrastructure and Islamic pilgrimage are suspected to influence the spectrum of MTBC lineages associated with EPTB infections in Saudi Arabia. Previous reports confirmed presence of almost all defined lineages including indigenous strains, but with no detailed analysis to elucidate genotyping and demographical determinates of EPTB infection [17–19]. Therefore, we aimed to conduct a cross-sectional study to investigate the impact of current demographical and ethnicity-related risk factors to EPTB disease characteristics among adolescents and adults in Saudi Arabia.

## Materials and methods

### Study population

A collection of 902 extrapulmonary MTBC *(M*.*tuberculosis*, *M*.*bovis*, *M*.*africanum*) culture isolates was obtained from four major referral hospitals during the study period of August 2014 to July 2016. This study was reviewed and approved by the Research Advisory Council at King Faisal Specialist Hospital and Research Centre, Riyadh. Clinical and demographical data were collected using standard data extraction forms. All data collected for the study was anonymized and no patient identifiers were used throughout the data collection process and analysis period. Cases with combined pulmonary and extrapulmonary infection, miliary TB, as well as pediatric EPTB (below 10 years) cases were excluded from study population.

### Sample collection, processing, and identification

MTBC culture isolates from EPTB cases were collected from four main referral centers in Riyadh: King Faisal Specialist Hospital and Research Centre, King Fahd Medical City, Prince Sultan Military Medical City and King Abdulaziz Medical City. All isolates were cultured on Lowenstein-Jensen’s media slants and were transferred to KFSHRC for genotyping analysis. Genomic DNA was extracted using the PrepIT MAX kit (DNA Genotek, Ottawa, Canada) as per the manufacturer’s instructions. Primary genotyping was carried out using the commercially available spoligotyping kit (Ocimum Biosolutions, Hyderabad, India). The 24-loci MIRU-VNTR typing based on quadruplex PCR (Genoscreen, Lille, France) was carried out according to the manufacturer’s instructions in a 3730xl DNA Analyzer (Life Technologies, CA, USA). Spoligotyping membranes were scanned and data were converted into numerical octal codes. Meanwhile, the alleles of MIRU-VNTR types were identified by the Genemapper version-4.0 (Applied Biosystems, CA, USA). Spoligo-octal signatures and the MIRU-VNTR allele profiles were both combined and submitted to the international online MIRU_VNTR database (www.miru-vntrplus.org) to assign lineages based on best-match and phylogenetic tree analysis.

### Data analysis

**Fig 1:**
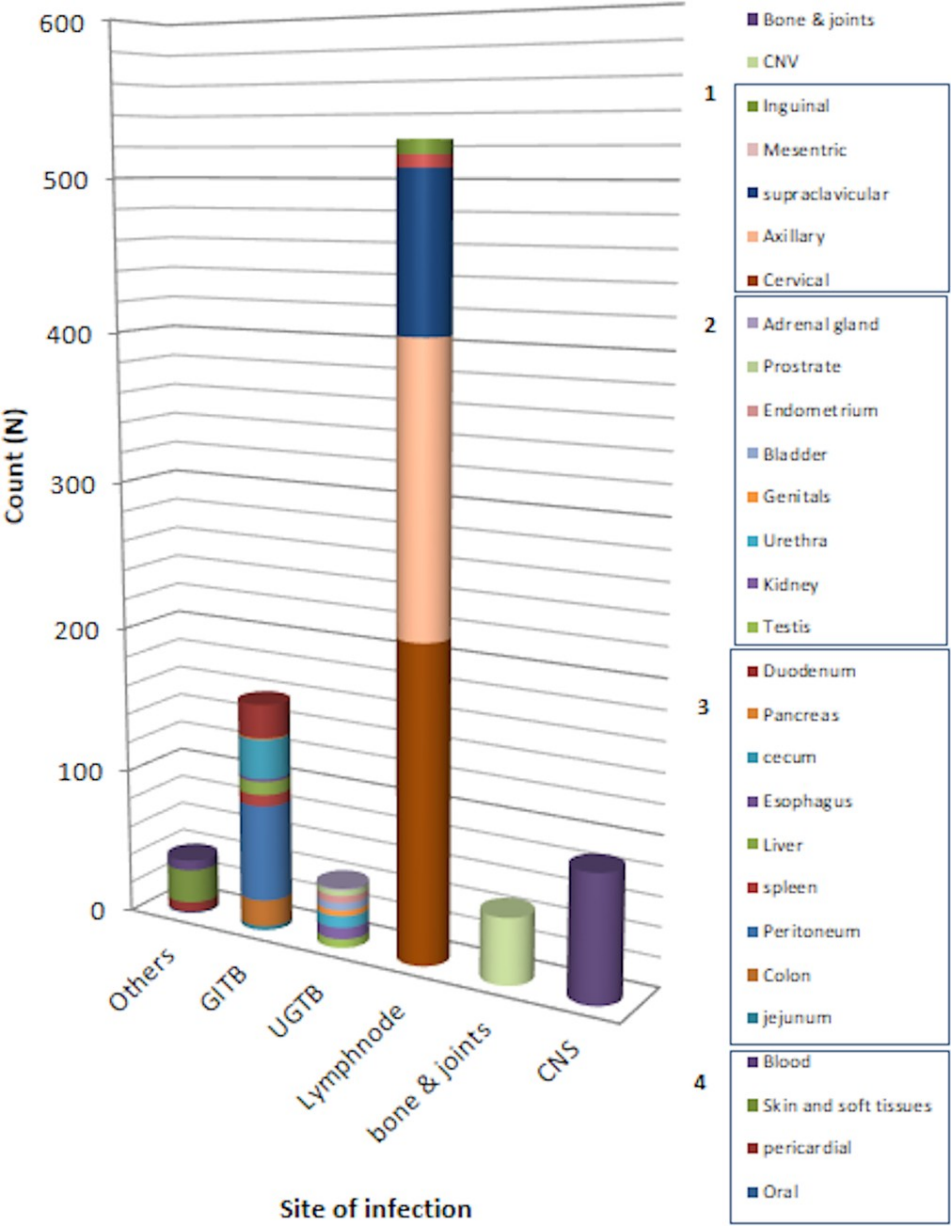
Sites of EPTB detected in this study. This figure illustrates the main sites of infection. Assigned numbers corresponds with subgroups of 1) lymph node TB, 2) urogenital TB, 3) gastrointestinal TB and 4) other TB infections.

Enrolled extrapulmonary cases were classified into six groups based on site of infection. These groups are lymph node, gastrointestinal, central nervous systems, bone and joints and urogenital. All other sites with lower proportions were grouped as “others” (Fig 1). The study population was further divided into Saudis and non-Saudis based on nationality. To study the impact of race/ethnicity, non-Saudis were further sub-grouped into six main categories such as European, American, African, Middle Eastern, South Asian, and South East Asian. *M*.*tuberculosis* lineages were defined into 6 major lineages according to large sequence polymorphisms based classification as Indo-Oceanic, East Asian, East African Indian, Euro-American, West African I and West African II [20].

Statistical analysis was carried out using SPSS version-20 package (IBM, NY, USA). The associations between categorical variables were examined by Chi-square test or fisher exact test. The strength of association between clinical and demographical variables was presented with odd ratios (OR) and 95% confidence interval (CI). In addition, all the variables with p-values less than 0.10 in bivariate or univariate analysis were included in multivariate logistic regression model analysis. All tests were two-tailed, and p-values less than 0.05 were considered statistically significant.

## Results

### Study population

Male patients (56.7%) were predominantly observed in this study, compared to female population (43.3%). Most of the enrolled patients were aged 35–60 years (34.6%), followed by 30.9% aged 25–35 years. The majority (83.4%) of cases was Saudis and only 16.6% was non-Saudis. Sub-classifying non-Saudi group into different origins showed a predominance of cases of South East Asian (44%) and South Asian (29.3%) origins. HIV positivity was limited only 4 (0.44%) cases. Demographical and clinical characteristics of all enrolled cases are summarized in Table 1.

**Table 1:** Demographical and clinical summary of 902 EPTB cases.

Parameters	Total cases/ %
**Gender**	
**Male**	511 (56.7)
**Female**	391 (43.3)
**Age groups**	
**Adolescence (10–18 years)**	62 (6.9)
**Young adulthood (19–24 years)**	155 (17.2)
**Early adulthood (25–34 years)**	279 (30.9)
**Middle adulthood (35–60 years)**	312 (34.6)
**Elderly (>60 years)**	94 (10.4)
**Nationality**	
**Saudi**	752 (83.4)
**Non-Saudi/Origins**	150 (16.6)
** European**	5 (3.3)
** American**	1 (0.7)
** African**	31 (20.7)
** South East Asian**	66 (44)
** Middle East**	3 (2)
** South Asian**	44 (29.3)
**AFB Smear**	
**Positive**	325 (36)
**Negative**	577 (64)
**HIV**	
**Positive**	4 (0.44)
**Negative/not available**	898 (99.6)
	

### Diversity of extra-pulmonary site of infection and MTBC lineages

**Fig 2:**
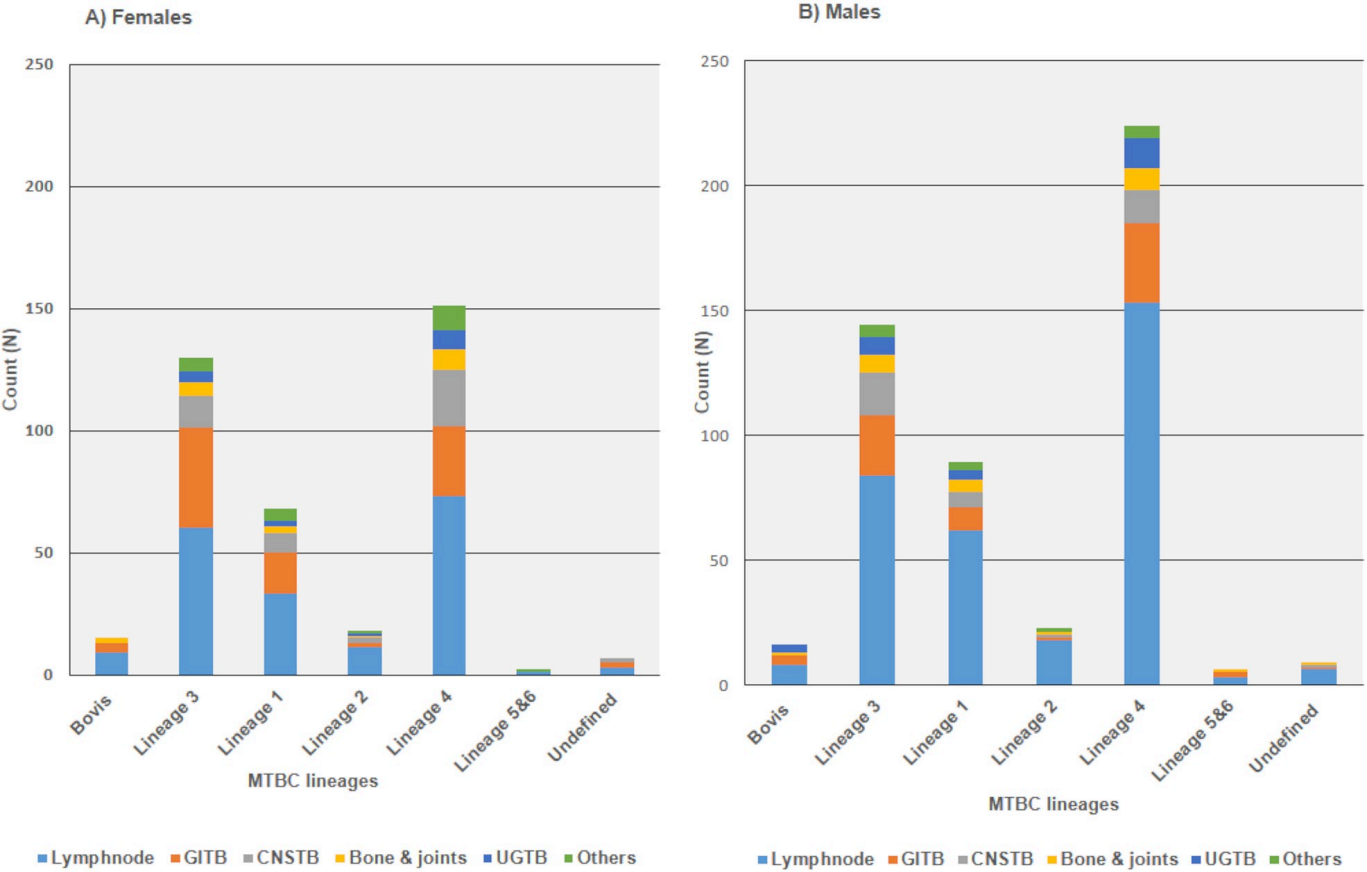
Gender, site of infection and MTBC lineages. Gastrointestinal TB (GITB), Central nervous System TB (CNSTB) and Urogenital TB (UGTB) were abbreviated as illustrated. Pericardial, skin and soft tissue, blood and oral TB were all grouped as others.

**Fig 3:**
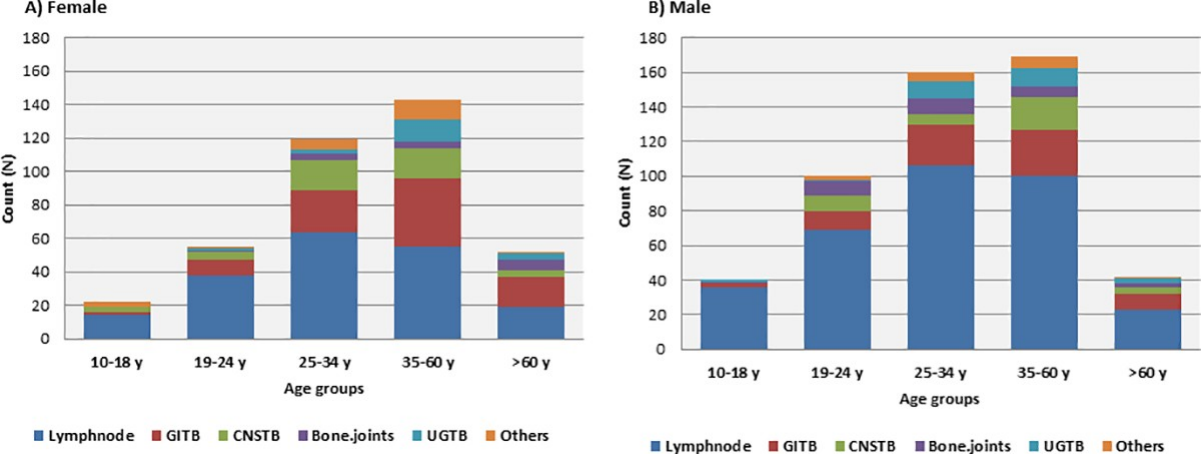
Gender, site of infection and distribution of age groups. Gastrointestinal TB (GITB), Central nervous System TB (CNSTB) and Urogenital TB (UGTB) were abbreviated as illustrated. All cases of pericardial, skin, soft tissue, blood and oral TB were represented as “Others”.

Lymph node (58.1%) was the most commonly reported site of infection followed by gastrointestinal system (18.7%), central nervous system (9.6%), bone and joints (5%) and urogenital systems (4.5%) respectively. There were different rare infection-sites such as blood, pericardial, oral, skin and soft tissues that together comprised 4.5% of the total enrolled cases. Detailed view of the main sites of infections is depicted in (Fig 1). Phylogenetic analysis of isolates with combined spoligotyping and MIRU-VNTR showed following MTBC lineage distribution;375(41.6%) of Euro-American (Lineage-4), 274(30.4%) of Central Asian (Lineage- 3), 157(17.4%) of Indo-oceanic (Lineage-1), 42(4.6%) of East Asian (Lineage-2), 5(0.5%) of West African I (Linegae-5), 2(0.2%) of West African 2 (Lineage -6) and 16(1.9%) of undefined lineages. In addition, 31(3.4%) of *M*. *bovis* strains were also identified among the study isolates. Distribution of major infection sites and MTBC lineages was very similar among male and female populations; lymph node presented as the main site of infections for both genders (Fig 2). Likewise, similar distribution of major infection sites was identified among different age groups (Fig 3).

### Association of demographic factors and EPTB disease characteristics

We analyzed the association of demographic data against major site of infections among adolescents and adults in Saudi Arabia. The results showed significant association between gender and site of infection (S1 Table). Male and female gender was significantly associated with lymph node TB (p<0.01; OR 1.99, 95%CI 1.52–2.61; p<0.01, OR 0.50, 95% CI 0.38–0.65), gastrointestinal TB (p<0.01; OR 0.52, 95% CI 0.37–0.73; p<0.01, OR 1.92, 95%CI 1.37–2.70), central nervous system TB (p 0.01; OR 0.57, 95% CI 0.37–0.90; p 0.01, OR 1.74, 94% CI 1.11–2.72) and urogenital TB (p<0.01; OR 0.42, 95%CI 0.22–0.81; p<0.01 OR 2.35, 95% CI 1.23–4.51) respectively. Lymph node TB, was primarily associated with adolescents (p<0.01; OR 3.21, 95%CI 1.68–6.13) and young adults (p<0.01; OR 1.76, 95% CI 1.22–2.55). In contrast, patients aged above 60 years have significant association for gastrointestinal TB (p<0.01; OR 1.91, 95% CI 1.18–3.1). The occurrence of TB of the bone and joints were significant among age group 35–60 years (p 0.02 OR 2.05, 95% CI 1.13–3.75). Nationality of the patient showed a significant association between (I) lymph node TB and non-Saudis (p<0.01; OR 1.87, 95% CI 1.27–2.73) and (II) gastrointestinal TB and Saudis (p<0.01; OR 5.76, 95% CI 3.32–10.0). In addition, South East Asian ethnicity showed a significant association with lymph node TB (p<0.01; OR 2.19, 95% CI 1.24–3.86) (S1 Table).

To accurately identify independent demographic risk factors significantly contributing to the disease characteristics of EPTB (site of infection and infecting MTBC genotypes) a bivariate analysis and subsequently multivariate regression model analysis of all significant factors were performed. Our results confirmed that Male-gender is an independent risk factor to urogenital TB (p 0.03; OR 2.77, 95% CI 1.11–6.96) and lymph node TB (p 0.005; OR 2.62, 95% CI 1.33–5.17) (Table 2).

**Table 2:** Association of demographical and clinical risk factors and EPTB-site of infection.

Variables	Total/%	Bivariate Analysis on sub-factorial level										Multivariate Analysis on sub-factorial level									
Site of infection	902(100)	Lymphonode		Gastrointestinal		Central nervous system		Bone and joints		Urogenital		Lymphonode		Gastrointestinal		Central nervous system		Bone and joints		Urogenital	
		OR (95% CI)	P value	OR (95% CI)	P value	OR (95% CI)	P value	OR (95% CI)	P value	OR (95% CI)	P value	OR (95% CI)	**P value**	**OR (95% CI)**	**P value**	**OR (95% CI)**	**P value**	**OR (95% CI)**	**P value**	**OR (95% CI)**	**P value**
Gender *																					
**Male**	511(56.7)	1.76(1.47, 2.10)	<0.01	0.77(0.57, 1.04)	0.09	2.53(1.39, 4.61)	<0.01	1.25(0.694, 2.25)	0.457	1.73(0.92, 3.27)	0.09	**2.62(1.33, 5.17)**	**0.005**	1.23(0.59, 2.53)	0.58	1.22(0.56, 2.66)	0.62	2.04(0.85, 4.93)	0.11	**2.77(1.11, 6.96)**	**0.03**
**Female**	391(43.3)	REF		REF		REF		REF		REF		REF		REF		REF		REF		REF	
Nationality*																					
**Non-Saudi**	150(16.6)	REF		REF		REF		REF		REF		REF		REF		REF		REF		REF	
**Saudi**	752(83.4)	3.943(3.17, 4.88)	<0.01	10.20(6.00,17.33)	<0.01	4.38(2.54, 7.53)	<0.01	10.25(3.67,28.62)	<0.01	12.67 (3.91, 41.0)	<0.01	0.90(0.36, 2.26)	0.82	1.78(0.63, 5.05)	0.28	0.81(0.28, 2.32)	0.70	1.57(0.40, 6.19)	0.52	2.57(0.58, 11.45)	0.22
Age *																					
**10–18**	62(6.9)	1.19(0.79, 1.79)	0.41	0.15(0.05, 0.42)	<0.01	0.38(0.1,1.41)	0.15	0.14(0.18, 1.161)	0.069	0.13(0.02, 1.00)	0.05	0.66(0.10, 4.24)	0.66	**0.11(0.013, 0.88)**	**0.04**	0.23(0.02, 2.17)	0.20	0.10(0.01, 1.54)	0.10	0.09(0.01, 1.41)	0.09
**19–24**	155(17.2)	2.55(1.78, 3.64)	<0.01	0.74(0.42,1.32)	0.31	1.750(0.73,4.17)	0.21	0.29(0.06, 1.37)	0.12	1.13(0.43,2.92)	0.81	1.40(0.22, 8.85)	0.72	0.53(0.08, 3.50)	0.51	1.08(0.145, 8.01)	0.94	0.18(0.02, 2.02)	0.17	0.74(0.10, 5.71)	0.77
**25–34**	279(30.9)	4.05(2.89,5.67)	<0.01	1.82(1.14,2.90)	0.01	3.00(1.35,6.7)	<0.01	1.71(0.68,4.35)	0.26	1.63(0.67,3.92)	0.28	0.65(0.14, 309)	0.59	0.35(0.07, 1.71)	0.20	0.52(0.09,2.87)	0.45	0.31(0.05, 1.84)	0.196	0.30(0.051, 1.72)	0.18
**35–60**	312(34.6)	3.69(2.62,5.19)	<0.01	2.52(1.16, 3.93)	<0.01	4.63(2.15, 9.93)	<0.01	3.29(1.41,7.66)	<0.01	1.25(0.49,3.17)	0.64	0.35(0.08, 1.58)	0.17	0.27(0.6, 1.23)	0.09	0.47(0.09, 2.45)	0.37	0.33(0.06,1.79)	0.20	0.12(0.02, 0.702)	0.02
**>60**	94(10.4)	REF		REF		REF		REF		REF		REF		REF		REF		REF		REF	
**Ethnicity**																					
**Saudi**	752(83.4)	REF		REF		REF		REF		REF											
**South Asian**	44(29)	1.88(0.43,8.07)	0.4	0.73(0.15,3.69)	0.71	0.91(0.16, 5.25)	0.92	1.17(0.184,7.43)	0.84	0.42(0.04, 4.9)	0.49										
**South East Asian**	66(44)	0.77(0.22,2.65)	0.67	0.21(0..04,1.1)	0.06	0.91(0.22,3.89)	0.90	-	-	0.56(0.09,3.657)	0.54										
**African**	31(20.7)	1.53(0.2, 11.78)	0.68	0.84(0.09,7.74)	0.88	2.29(0.26,20..37)	0.46	0.78(0.47, 12.97)	0.86	-	-										
**European**	5(3.3)																				
**American**	1(0.7)																				
**Middle Eastern**	3(2)																				

*Factors with an overall p value <0.10 in bivariate analysis were included in multivariate regression model analysis. Reference subgroup for “site of infections” was “others”.

### Demographical and clinical risk factors

The second primary aim of this paper was to identify demographic (age, gender, nationality and origin) and clinical factors (infecting lineage and site of infection) significantly associated with EPTB. A bivariate analysis was performed for all factors among 902 EPTB cases, stratified into two different age groups: adolescents and adults (Table 3). Significant (P>0.1) demographic and clinical factors in a bivariate analysis were accordingly included in a multivariate regression model analysis (Table 3).

**Table 3:** Association of demographical and clinical risk factors among adult and adolescents EPTB patients.

Variables	Total/%	Adolescent n = 62	Adults n = 840	Bivariate Analysis		Multivariate Analysis	
				OR (95% CI)	P value	OR (95% CI)	P value
**Gender**							
Male	511(56.7)	40(64.5)	471(56.1)	REF			
Female	391(43.3)	22(35.5)	369(43.9)	1.42(0.83–2.44)	0.19	0.59(0.39–0.92)	0.30
**Nationality**							
Non-Saudi	150(16.6)	22(35.5)	128(15.2)	REF			
Saudi	752(83.4)	40(64.5)	712(84.8)	3.05(1.76–5.32)	**<0.01**	**0.16(0.05–0.53)**	**0.002**
**Ethnicity**							
Saudi	752(83.4)	40(64.5)	712(84.8)	REF			
South Asian	44(29)	9(14.5)	35(4.2)	0.22(0.09–0.48)	**<0.01**	4.98(.67–36.84)	**0.005**
South East Asian	66(44)	6(9.7)	60(7.1)	2.57(0.84–7.83)	0.09	8.64(1.18–63.16)	**0.03**
African	31(20.7)	6(9.7)	25(2.9)	1.07(0.34–3.39)	0.91	3.51(0.47–26.31)	0.22
European	5(3.3)	1(1.6)	4(0.50)				
American	1(0.7)		1(0.11)				
Middle Eastern	3(2)	-	3(0.35)				
**HIV status**							
Positive	4(0.44)	-	4(0.50)	REF			
Negative/Unknown	898(99.5)	62(100)	836(99.5)	1.49(0.07–27.93)	0.79	0.81(0.07–8.79)	0.86
**AFB Smear**							
Positive	325(36.1)	21(33.9)	304(36.2)	REF			
Negative	577(63.9)	41(66.1)	536(63.8)	0.90(0.52–1.55)	0.71	0.91(0.58–1.42)	0.66
**MTBC Lineages**							
*M*.*bovis*	31(3.4)	2(3.2)	29(3.4)	REF			
Lineage-1 Indo-Oceanic	157(17.4)	5(8.1)	152(18.2)	2.09(0.39–11.33)	0.39	0.68(0.15–3.13)	0.61
Lineage-2 East Asian	42(4.7)	4(6.4)	38(4.5)	0.65(0.11–3.82)	0.64	0.86(0.13–5.54)	0.65
Lineage-3 East African Indian	274(30.4)	18(29)	256(30.5)	0.98(0.22–4.44)	0.98	0.44(0.1–1.92)	0.27
Lineage-4 Euro American	375(41.6)	33(53.2)	342(40.7)	0.71(0.16–3.12)	0.65	0.63(0.14–2.76)	0.54
Lineage -5 West African -I	5(0.5)	0	5(0.60)	0.93(0.04–22.19)	0.96	0.29(0.02–4.07)	0.36
Lineage-6 West African II	2(0.2)	0	2(0.24)	0.42(0.01–11.48)	0.61	-	
‘Undefined’	16(1.8)	0	16(1.90)	2.79(0.12–61.8)	0.51	-	
**Site of infection**							
Others	38(4.2)	3(4.8)	35(4.2)	REF			
Lymphnode	524(58.1)	50(80.7)	474(56.4)	0.81–0.24–2.73)	0.74	0.62(0.14–2.69)	0.53
Gastrointestinal	168(18.6)	4(6.5)	164(19.5)	3.51(0.75–16.40)	0.11	0.31(0.07–1.36)	0.12
Central nervous system	86(9.5)	3(4.8)	83(9.9)	2.37(0.46–12.32)	0.30	0.57(0.11–2.82)	0.49
Bone and joints	45(5)	1(1.6)	44(5.2)	3.77(0.37–37.85)	0.26	0.32(0.06–1.65)	0.17
Urogenital	41(4.6)	1(1.6)	40(4.8)	3.43(0.34–34.48)	0.29	0.24(0.05–1.23)	0.09

Factors showed p value <0.10 in bivariate analysis were included in the multivariate regression analysis. Adolescents group was excluded from the analysis due to very low number in presentation.

South Asian (p 0.01; OR 4.98, 95% CI 0.67–36.84) and South East Asian (p 0.03; OR 8.64, 95% CI 1.18–63.16) ethnicities were identified with a significant association with EPTB. Surprisingly, however, none of the remaining demographic and clinical factors showed significant association for EPTB, including infecting MTBC lineages (Table 3).

## Discussion

Limited evidence exists in literature when it comes to the impact of demographic factors on EPTB disease characteristics globally and particularly in Saudi Arabia [2–4]. Therefore, the primary objective of this cross-sectional study was to investigate the impact of demographical and clinical factors on EPTB disease characteristics among adolescents and adults in the country.

Our demographic data showed predominance of male gender, Saudi nationals and patients aged 25 to 34 years and 36 to 60 years (Table 1). Predominance of males at their early and middle adulthoods comes in concordance to what has been previously reported in several studies from Saudi Arabia [16–18, 21]. Meanwhile, deterioration of immune status, due to environmental, social as well as genetic factors (e.g inherited, or epigenetic influences), might have played a role in increasing the risk of aging males to EPTB infections. Contrary to these findings, several reports showed a particular prevalence of female gender among EPTB as well as PTB infections [2–4]. The discrepancy in frequency and distribution of gender in our findings and the latter studies could be due to socio-economic and cultural aspects as well as overall lifestyle habits of individuals. For instance, the conservative culture of native Saudi population might ultimately limit risk of exposure of females to infectious bacilli [22–24]. In contrast, Saudi men have higher social, financial and mobility freedom. Subsequently, they may experience higher risk of exposure to infectious agents. Future qualitative research remains primitive in providing clues to the underlying reasons of male risk to EPTB infections in Saudi Arabia.

Regression analysis of demographical factors against site of EPTB infections showed male-gender as an independent risk factor to lymph node TB (Table 2). While similar findings have been recently established in Saudi Arabia on a national scale [25, 26], several previous reports documented increased risk of females to lymph node TB in different geographical regions [27–31]. Variations in immune-related factors (e.g. levels of tumor necrosis factor (TNF-alpha) and interleukin-10 (IL-10) as well as CD4+ counts) between both genders could be a reasonable factor for increased male susceptibility to TB lymphadenitis [32, 33]. Other plausible aspects include variability in endocrine, socio-economic and cultural aspects experienced by different genders in each population. In addition, consistent with previous reports in the literature [34, 35], males were also presented with a particular risk to urogenital TB (Table 3). However, it is important to note that incidence of female-urogenital TB (e.g. genital TB) is believed to be largely and globally undermined especially among single females and those not at their reproductive age. Wherein, TB of the female genital organs is associated with infertility, menstrual irregularities, dyspareunia, and chronic pelvic inflammatory diseases, which explains why such cases are often suspected during evaluation of infertility among females at their reproductive age [36, 37]. In line with this possibility, 60% of identified female-urogenital TB cases in this study population were at their reproductive age (22 to 46 years old) (Fig 2). Whereas male-urogenital TB cases were more distributed among all age groups (constituting more than 40% of each age group) (Fig 2).

Adolescents’ cases showed statistically significant association with lymph node TB (p<0.01; OR 3.21, 95%CI 1.68–6.13). Similarly, previous studies also demonstrated higher risk of adolescents to EPTB and mainly lymph node TB [38–40]. On the other hand, association of young adults to lymph node TB is a rare finding in the Kingdom. A recent national study showed low risk for individuals aged below 25 years to EPTB [25]. However, the risk of young adults to EPTB was well established in other regions, particularly to lymph node TB, gastrointestinal and osteoarticular TB [4, 41]. In contrast, older patients (>60 years) have higher risk of gastrointestinal TB and similar finding has been reported from other countries [4]. Despite the latter associations, multivariate regression model analysis revealed no significant impact of age to disease characteristics of EPTB, including site of infection (Table 2, Table 3). This is rather an interesting finding, considering that aging is known to cause significant decline in immune status and hence increased susceptibility to both TB and EPTB infections [42–44]. It remains highly possible that the actual impact of aging was largely undermined in our settings due to high consanguinity rate (~60%) in the population [45]. High consanguinity rate may result in overall low immune status of subjects of all age groups (due to high predisposal of homozygotes deleterious mutations), a phenomenon that could explain the potential representation of ageing as a “silent risk factor” for EPTB.

The exact role of host and microbial factors contributing to TB clinical manifestation remain to a large extent controversial [46, 47]. Cumulative evidence suggested that patient’s ethnicity plays a critical role in determining TB clinical phenotype [48]. Concordant with this view, we revealed significant association between patients’ nationality and several sites of infections (S1 Table). Particularly, we found that Saudi as well as non-Saudi patients have higher risk of developing gastrointestinal and lymph node TB respectively (S1 Table). In addition, several ethnicities were also identified as independent risk factors for EPTB (Table 3). Few fairly recent studies suggested that ethnicity-related factors (e.g. polymorphisms and haplotypes) play a significant role in modulating host immune responses during MTBC infections [49]. In one of these studies, authors reported different inflammatory profiles (e.g neutrophil counts, serum concentrations of CCL2, CCL11, CCL5, IL-1 and IL-12) among African ancestry-patients versus that of Eurasian ancestry in London population with no significant impact of lineage diversity on clinical presentation of TB [46].

While EPTB burden is globally and synergistically increasing with the global rise of HIV infections, little is known regarding the main demographical risk factors to EPTB in moderate-TB and low-HIV burden settings, exemplified by Saudi Arabia. The features of the current study provide a great opportunity to study independent demographical risk factors to disease characteristics of EPTB for the first time in the country. However, the study has few limitations. Lack of detailed information regarding lifestyle habits of patients (cigarette smoking and exercising), clinical information (previous TB infection, clinical symptoms and the existence of other comorbidities) and living standards (rural places, travelling activities and socio-economical standards) restricted us from performing further analysis.

## Conclusion

Gender and ethnic origins have been identified as independent risk factors for EPTB among adolescents and adults in Saudi Arabia. However future, nationwide qualitative studies are highly needed to further understand the main triggers and trends of host-pathogen interactions and, thus, improve the overall clinical management and diagnosis of EPTB cases in Saudi Arabia.

## Supporting information

S1 Table Frequency of demographic factors and site of EPTB infection. (DOCX)
