# Supplementary material for: Demographic risk factors for extra-pulmonary tuberculosis among adolescents and adults in Saudi Arabia
Source: PLoS One. 2019 Mar 27;14(3):e0213846. doi: 10.1371/journal.pone.0213846 (PMC6436801; doi:10.1371/journal.pone.0213846)
Supplement: S1 Table — (DOCX) [file pone.0213846.s001.docx]

***S1 Table: Frequency of Demographic factors and site of EPTB infection.***

| Variables | Total/%  N=902 | Lymphnode  N=524 | OR (95% CI); P value | GITB  N=168 | OR (95% CI); P value | CNSTB  N=86 | OR (95% CI); P value | Bone/Joints  N=45 | OR (95% CI); P value | UGTB  N=41 | OR (95% CI); P value | Others  N=38 |
| --- | --- | --- | --- | --- | --- | --- | --- | --- | --- | --- | --- | --- |
| **Gender** |  |  |  |  |  |  |  |  |  |  |  |  |
| Male | 511(56.7) | 334(63.7) | 1.99(1.52-2.61); <0.01 | 73(43.5) | 0.52(0.37-0.73); <0.01 | 38(44.2) | 0.57(0.37-0.90); 0.01 | 25(55.6) | 0.95(0.52-1.74); 0.88 | 15(36.6) | 0.42(0.22-0.81); <0.01 | 15(39.5) |
| Female | 391(43.3) | 190(36.3) | 0.500(0.38-0.65); <0.01 | 95(56.5) | 1.92(1.37-2.70); <0.01 | 48(55.8) | 1.74(1.11-2.72); 0.01 | 20(44.4) | 1.05(0.57-1.92); 0.87 | 26(63.4) | 2.35(1.23-4.51), <0.01 | 23(60.5) |
| **Age Groups** |  |  |  |  |  |  |  |  |  |  |  |  |
| 10-18 | 62(6.9) | 50(9.6) | 3.21(1.68-6.13); <0.01 | 4(2.3) | 0.28-0.10-0.79); 0.11 | 3(3.5) | 0.46(0.14-1.51); 0.20 | 1(2.2) | 0.30(0.04-2.19); 0.23 | 1(2.4) | 0.33(0.04-2.42); 0.27 | 3(7.9) |
| 19-24 | 155(17.2) | 107(20.4) | 1.76(1.22-2.55); <0.01 | 20(11.9) | 0.60(0.36-0.99); 0.04 | 14(16.3) | 0.93(0.51-1.96); 0.81 | 2(4.4) | 0.21(0.05-0.89); 0.03 | 9(22) | 1.38(0.64-2.94); 0.41 | 3(7.9) |
| 25-34 | 279(30.9) | 170(32.4) | 1.18(0.89-1.58); 0.25 | 49(29.2) | 0.90(0.62-1.3); 0.58 | 24(27.9) | 0.85(0.52-1.39); 0.52 | 12(26.7) | 0.80(0.41-1.58); 0.53 | 13(31.7) | 1.03(0.53-2.04); 0.91 | 19(50) |
| 35-60 | 312(34.6) | 155(29.6) | 0.59(0.45-0.78); 0.0002 | 68(40.5) | 1.37(0.97-1.92); 0.08 | 37(43) | 1.48(0.94-2.33); 0.08 | 23(51.2) | 2.05(1.13-3.75); 0.02 | 10(24.4) | 0.59(0.29-1.23), 0.16 | 12(31.6) |
| >60 | 94(10.4) | 42(8) | 0.55(0.35-0.84); <0.01 | 27(16.1) | 1.91(1.18-3.1); <0.01 | 8(9.3) | 0.87(0.41-1.86); 0.72 | 7(15.5) | 1.63(0.71-3.76); 0.25 | 8(19.5) | 2.18(0.98-4.89); 0.06 | 1(2.6) |
| **Nationality** |  |  |  |  |  |  |  |  |  |  |  |  |
| Saudi | 752(83.4) | 418(79.8) | 0.52(0.36-0.76); <0.01 | 153(91.1) | 5.76(3.32-10.0); <0.01 | 70(81.4) | 0.86(0.48-1.52); 0.60 | 41(91.1) | 2.10(0.74-5.97); 0.16 | 38(92.7) | 2.6(0.79-8.56); 0.11 | 32(84.2) |
| Non-Saudi | 150(16.6) | 106(20.2) | 1.87(1.27-2.73); <0.01 | 15(8.9) | 0.06(0.03-0.09); <0.01 | 16(18.6) | 1.16(0.65-2.06); 0.60 | 4(8.9) | 0.47(0.17-1.35); 0.16 | 3(7.3) | 0.38(0.11-1.25); 0.11 | 6(15.8) |
| **Ethnicity** |  |  |  |  |  |  |  |  |  |  |  |  |
| South Asian | 44(29.3) | 30(28.3) | 1.528(0.85-3.02); 0.17 | 3(20) | 0.31(0.09-1.0); 0.05 | 6(37.6) | 1.53(0.63-3.74); 0.34 |  | - | 2(66.7) | 1.0(0.23-4.28); 1.0 | 3(50) |
| South East Asian | 66(44) | 49(46.3) | 2.19(1.24-3.86); <0.01 | 7(46.7) | 0.49(0.22-1.11); 0.08 | 4(25) | 0.59(0.21-1.97); 0.32 | 3(75) | 0.90(0.27-2.99); 0.86 | 1(33.3) | - | 2(33.3) |
| African | 31(20.7) | 20(18.9) | 1.32(0.62-2.79); 0.46 | 4(26.7) | 0.64(0.22-1.85); 0.41 | 5(31.2) | 1.87(0.70-5.01); 0.21 | 1(25) |  |  |  | 1(16.7) |
| European | 5(3.3) | 5(4.7) | 8.01(0.44-145-.38); 0.16 |  | - |  | - |  |  |  |  |  |
| American | 1(0.7) | 1(0.9) | - |  | - |  | - |  |  |  |  |  |
| Middle Eastern | 3(2) | 1(0.9) | - | 1(6.6) | - | 1(6.2) | - |  | - |  |  |  |
